# Case Report: Prenatal and postnatal clinical manifestations of 15q duplication syndrome: a single longitudinal case combined with pooled analysis of 35 prenatal cases

**DOI:** 10.3389/fmed.2026.1928018

**Published:** 2026-10-15

**Authors:** Ziqing Xu, Mou Ufei, Hongbo Chen, Shuo Yang, Peijun Zou, You Lu, Fei Xiong, Jinrong Li

**Affiliations:** 1 Department of Pediatrics, West China Second University Hospital, Sichuan University, Chengdu, China; 2 Key Laboratory of Birth Defects and Related Diseases of Women and Children, Ministry of Education, Sichuan University, Chengdu, Sichuan, China; 3 West China Medical College, Sichuan University, Chengdu, Sichuan, China

**Keywords:** 15q duplication syndrome, copy number variation, genetic counseling, non-invasive prenatal screening, prenatal diagnosis

## Abstract

**Background:**

15q duplication (Dup15q) syndrome is a rare neurodevelopmental disorder characterized by early-onset hypotonia, developmental delay, intellectual disability, symptoms related to autism spectrum disorder and epilepsy. In the absence of specific treatment, prenatal diagnosis is of important clinical significance. However, most reported cases are postnatal cases, whereas prenatal cases are limited and often lack detailed clinical information, making prenatal recognition of this disorder challenging. In this study, we integrated prenatal and postnatal clinical information from the present case with a pooled analysis of published prenatal Dup15q syndrome cases, providing additional insights into the prenatal-to-postnatal phenotypic spectrum of Dup15q syndrome.

**Case presentation:**

We report a case of Dup15q syndrome with both prenatal and postnatal clinical data. Although prenatal ultrasound showed polyhydramnios, an abdominal circumference below the 10th percentile, and mild tricuspid regurgitation, no further prenatal genetic testing was performed. After birth, the patient presented with early hypotonia and developmental delay, and the diagnosis was established by genetic testing after a poor response to rehabilitation. We collected 34 prenatal Dup15q syndrome cases through literature review. The results showed that non-invasive prenatal screening (NIPS) may facilitate the prenatal identification of Dup15q syndrome. Prenatal ultrasound findings included polyhydramnios, fetal growth restriction (FGR), cardiac abnormalities, dysmorphic features, and abnormal ultrasound soft markers. The pooled analysis further provided an overview of the prenatal phenotypic spectrum of Dup15q syndrome and suggested that FGR and polyhydramnios, particularly in the presence of additional ultrasound abnormalities, positive NIPS results, or a positive family history, may warrant further etiological evaluation.

**Conclusions:**

Dup15q syndrome exhibits prominent clinical heterogeneity with nonspecific prenatal manifestations, predisposing patients to missed or delayed diagnosis. By integrating prenatal and postnatal clinical information with pooled prenatal cases, this study provides a broader understanding of the phenotypic spectrum of Dup15q syndrome and may facilitate prenatal counseling and early clinical recognition.

## 1 Introduction

15q duplication (Dup15q) syndrome is a rare neurodevelopmental disorder first reported in 1993 (1). Since then, related cases have been reported worldwide, with an estimated incidence of approximately 1 in 30,000 (2). This syndrome results from one or more extra copies of the Prader-Willi/Angelman critical region (PWACR) within chromosome 15q11.2-q13.1 (3).

Dup15q syndrome exhibits significant clinical heterogeneity, with common manifestations including developmental delay, symptoms related to autism spectrum disorder (ASD), epilepsy, dysmorphic features, and behavioral difficulties (4) As no disease-specific treatment is currently available, early recognition and timely rehabilitation interventions are crucial for improving patient prognosis and quality of life (5).

Despite increasing recognition of this syndrome, prenatal clinical data are limited (6). Specific prenatal signs are often absent, prenatal identification of Dup15q syndrome remains challenging, and the currently available evidence is still insufficient to adequately guide prenatal diagnosis, genetic counseling, and pregnancy-related decision-making. Differing from previous studies that separately reported prenatal or postnatal cases, this study integrates a postnatally diagnosed proband with retrospectively collected prenatal findings and a pooled analysis of reported prenatal cases, allowing a broader characterization of the prenatal and postnatal phenotypic features of Dup15q syndrome.

Here, we report a case of Dup15q syndrome and review the relevant literature to deepen understanding of its prenatal clinical features and provide additional information for prenatal diagnosis and genetic counseling.

## 2 Case presentation

A 1-year-old girl presented to our hospital for evaluation of global developmental delay. She exhibited generalized hypotonia and could sit, but was unable to transition from supine to sitting, crawl, or pull to stand. She had not yet developed babbling such as “ba-ba” or “ma-ma”, did not respond to her name, and showed poor eye contact and absent social interaction.

### 2.1 Personal history

The girl was the second child of her mother and was delivered vaginally at 39 + 6 weeks, with the birth weight of 3.31 kg and the length of 50 cm. After birth, she was hospitalized for neonatal pneumonia, left temporoparieto-occipital subdural hemorrhage, and neonatal jaundice. During infancy, she had recurrent hospitalizations for respiratory tract infections and one febrile seizure. She also had poor sleep, increased fussiness, normal appetite, frequent choking with feeding or drinking, abdominal distension, and lactose intolerance.

### 2.2 Prenatal abnormalities

Second-trimester ultrasonic abnormalities: Fetal small abdominal circumference (＜10th percentile, suggestive of FGR); Polyhydramnios; Mild tricuspid regurgitation. Serum prenatal screening was low-risk, leading to ignored genetic alert. Further prenatal genetic testing [non-invasive prenatal screening (NIPS) and amniocentesis] was declined by family, resulting in missed prenatal diagnosis.

### 2.3 Family history

She had one healthy older brother aged 6 years. Her mother and father were 34 and 37 years old, respectively, at the time of delivery. The parents were healthy and non-consanguineous, and there was no family history of birth defects or genetic disease.

### 2.4 Physical examination

Her growth curves are shown in Figure 1. Growth parameters were generated according to the World Health Organization 2006 Child Growth Standards. At 1 year of age, her length was 72.3 cm (Z-score = −0.96), weight was 7.8 kg (Z = −1.27), and head circumference was 46.6 cm (Z = 1.12). She had frontal bossing, mild scaphocephalic features, and generalized hypotonia. No obvious abnormalities were noted on examination of the heart, lungs, abdomen, or external genitalia.

**Figure 1:**
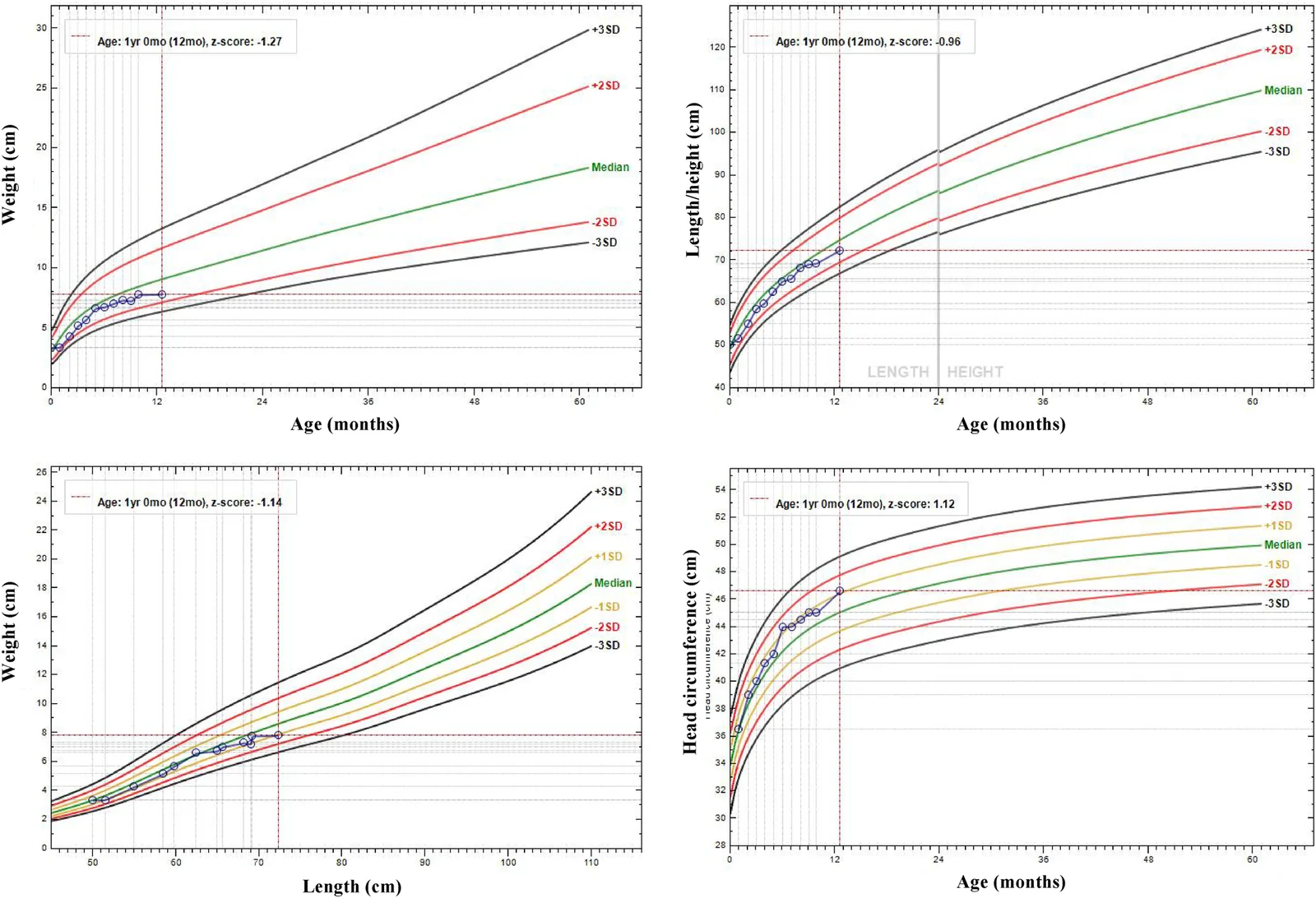
The growth chart of the patient from birth to 1 year of age, plotted according to the world health organization 2006 child growth standards.

### 2.5 Auxiliary examinations

Laboratory investigations, including complete blood count, liver and renal function tests, thyroid function tests, blood ammonia, lactate, and pyruvic acid were unremarkable, except for an elevated *β*-hydroxybutyrate level (2.06 mmol/L). Echocardiography revealed a patent foramen ovale. Brain MRI performed at 1 and 8 months of age showed no definite abnormalities.

### 2.6 Developmental milestones

She achieved rolling over at 5 months of age and was able to reach for and grasp objects at 7 months. At 9 months of age, she was still unable to maintain a stable sitting position even with support, crawl, or pull to stand. Assessment with the Bayley Scales of Infant Development showed a Mental Development Index (MDI) of <50 (<0.1%) and a Psychomotor Development Index (PDI) of <50 (<0.1%), indicating significant developmental delay. Rehabilitation therapy was initiated at approximately 9 months of age, for 30 min daily, and continued for 4 months; however, motor improvement was minimal. Independent sitting was achieved only at 12 months. At 13 months, she could produce nonspecific “ba-ba” and “ma-ma” sounds, but remained unable to crawl, pull to stand, or transition from supine to sitting.

### 2.7 Genetic findings

Trio-based whole-exome sequencing identified a copy number gain (4 copies, tetrasomy) involving 15q11.1-q13.1 (GRCh38/hg38, chr15:19964676_28712359), spanning 8.75 Mb (Figure 2). This duplication was considered *de novo*, as it was not identified in either parent. SNP-based analysis of the sequencing data suggested a maternal origin of the duplicated region.

**Figure 2:**
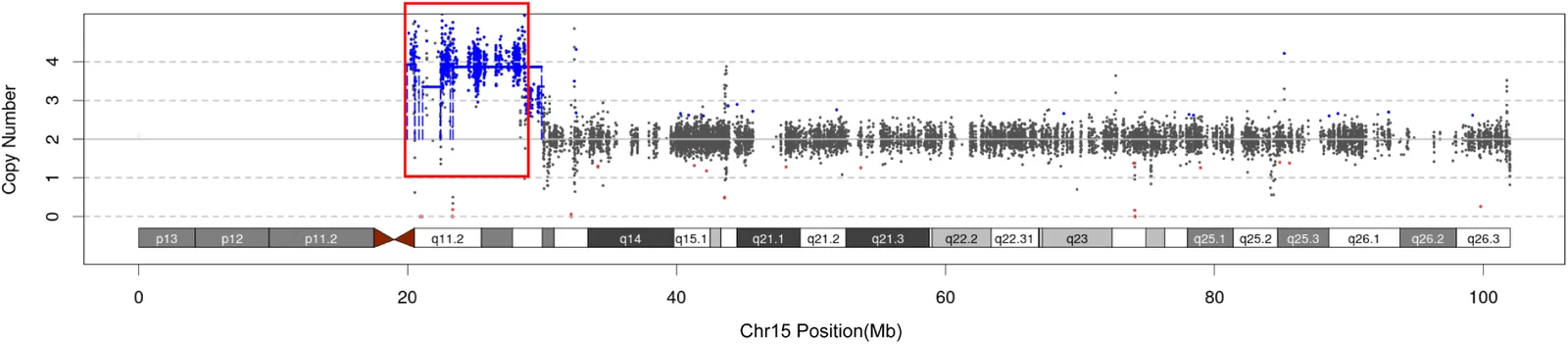
Whole-exome sequencing identified an 8.75-mb copy number variant in the 15q11.1-q13.1 region of the patient (GRCh38/hg38, chr15:19964676_28712359). The red box highlights the extent of the duplicated region.

## 3 Literature review

We searched PubMed, Web of Science and Embase from database inception to September 8, 2026, using combinations of keywords related to 15q duplication syndrome and prenatal presentation. The literature screening process is shown in Figure 3. Eligible cases were prenatal Dup15q cases with available clinical and genetic information. Cases were excluded if prenatal clinical information was unavailable, genetic information was insufficient to define the 15q11–q13 duplicated region or copy number change, or additional chromosomal abnormalities, CNVs, or monogenic variants were present. After screening, 12 publications were included, and one additional eligible study was identified through reference review. Detailed case information is provided in Supplementary Table S1 (6–18).

**Figure 3:**
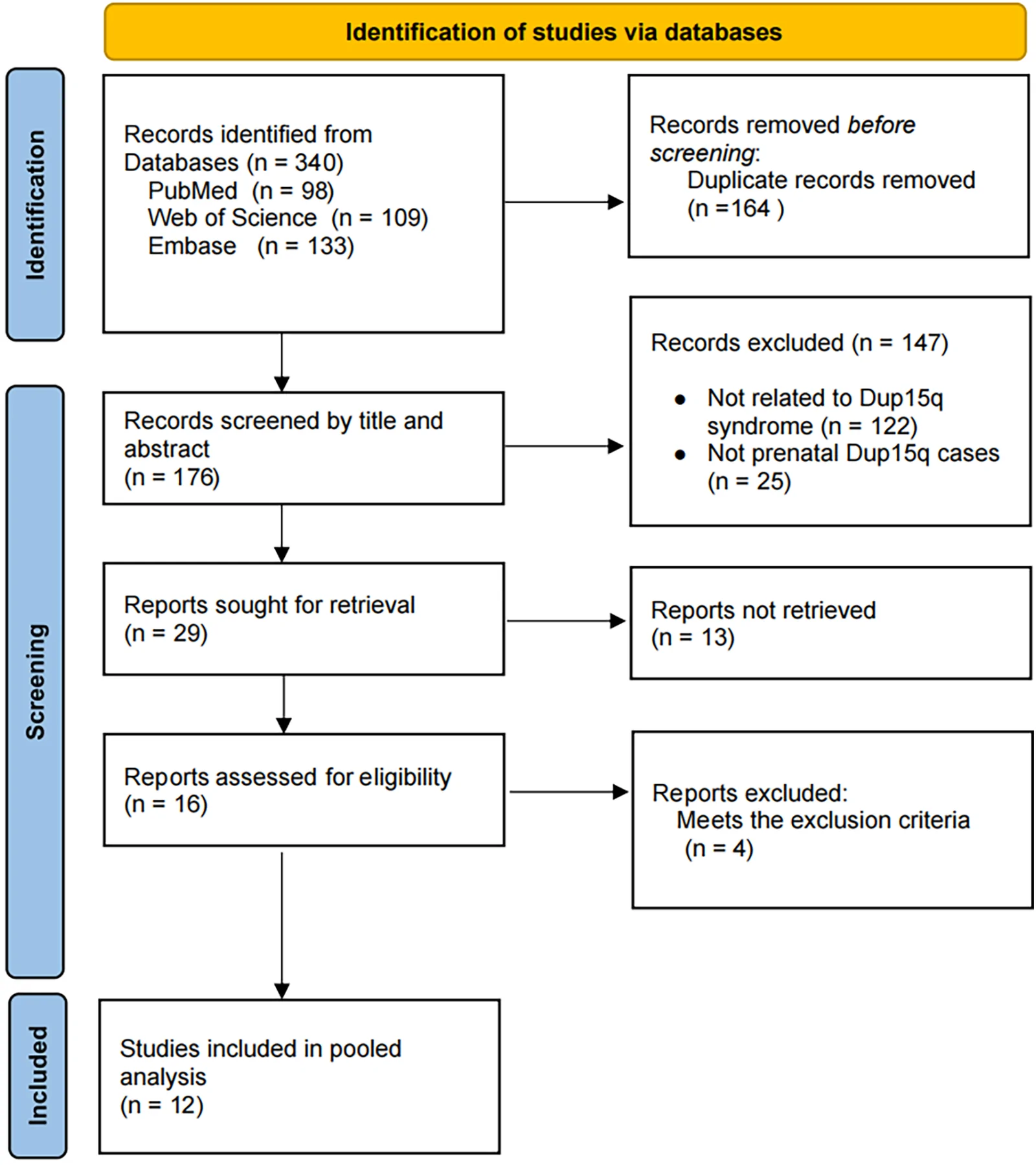
Flow diagram of the literature search and study selection process.

Among the 35 prenatal cases, including 34 previously reported cases and our case, 48.6% (17/35) involved advanced maternal age and 22.2% (6/27) had an adverse pregnancy history or abnormal family history. Based on the available prenatal ultrasound records (*n* = 29), we summarized the frequency of prenatal ultrasound findings in the pooled case series. Normal prenatal ultrasonography was observed in 62.1% (18/29) of cases, whereas ultrasound abnormalities were identified in 37.9% (11/29), including FGR, polyhydramnios, cardiac abnormalities, malformations, and abnormal soft markers (Table 1). Regarding prenatal screening, 20 cases underwent NIPS, all of which were classified as high risk. All abnormal karyotypes with available inheritance information were *de novo* in origin. Most families opted for termination of pregnancy (TOP) (63.6%, 21/33).

**Table 1:** Prenatal ultrasound findings of prenatal cases of 15q duplication syndrome.

Prenatal ultrasound findings	Case number	Percentage
Overall ultrasound findings (*n* = 35)		
Normal	18	51.4%
Abnormal	11	31.4%
NA	6	17.1%
Specific prenatal ultrasound findings (*n* = 29)^a^		
FGR, AC < P10	4	13.8%
Polyhydramnios	3	10.3%
Cardiac abnormalities		
VSD	2	6.9%
Dysmorphic features		
Facial dysmorphology	1	3.4%
Polydactyly	1	3.4%
Ultrasound soft markers		
Increased NT	1	3.4%
Renal pelvis separation	1	3.4%
Bilateral choroidal cysts	1	3.4%
Absent nasal bone	1	3.4%
Cerebral ventriculomegaly	1	3.4%
Echogenic intracardiac focus	1	3.4%
Number of Coexisting Abnormal Findings (*n* = 10)^b^		
1 abnormal finding	5	50.0%
2 abnormal findings	3	30.0%
3 abnormal findings	2	20.0%

AC, abdominal circumference; FGR, fetal growth restriction; NT, nuchal translucency; VSD, ventricular septal defect. ^a^Six cases without available prenatal ultrasound information were excluded from the analysis of specific prenatal ultrasound findings. ^b^For the analysis of the number of coexisting abnormal findings, cases with unavailable ultrasound data (*n* = 6), normal ultrasound findings (*n* = 18), and isolated placental/cord abnormalities (*n* = 1, case 6) were excluded.

## 4 Discussion

The chromosome 15q proximal region contains multiple low copy repeats (LCRs) that harbor five breakpoints (BP1-5), making it prone to chromosomal rearrangements (19). The PWACR is located between BP2 and BP3 within chromosome 15q11.2-q13.1. The PWACR is imprinted and deletions or duplications of this segment give rise to distinct clinical syndromes. Specifically, paternally and maternally derived deletions are associated with Prader-Willi syndrome and Angelman syndrome respectively, while duplications of this region are linked to Dup15q syndrome (20).

Dup15q syndrome is an autosomal dominant neurodevelopmental disorder characterized by early-onset hypotonia, developmental delay/intellectual disability, symptoms related to ASD, epilepsy, dysmorphic features and behavioral difficulties (20). The primary mechanisms are categorized into two types: a maternal isodicentric 15q11.2-q13.1 supernumerary chromosome (idic(15)), typically containing two extra copies and resulting in tetrasomy (∼60%–80%); and an interstitial 15q11.2-q13.1 duplication that typically involves one extra copy within chromosome 15, resulting in trisomy (∼20%–40%) (3, 21). To date, copy number variations (CNVs) in the 15q11-q13 segment have been observed to vary between 3 and 6 copies, corresponding to triplication, tetrasomy, pentasomy, and hexasomy, respectively. Rare cases of octasomy have also been reported (22). Previous studies have demonstrated an association between higher copy number gains and postnatal clinical severity (22); however, whether this relationship extends to prenatal manifestations remains unclear. In our pooled analysis, the only case with a 6-copy gain involving 15q11-q13 (case 23) exhibited multiple severe prenatal ultrasound abnormalities. However, no clear trend was observed across different copy-number groups, likely due to the limited number of cases with higher copy numbers (Table 2). These observations provide a hypothesis that warrants further validation in larger cohorts.

**Table 2:** Prenatal ultrasound findings according to 15q11-q13 copy number.

Prenatal ultrasound findings	15q11-q13 Copy number				
	2–3 copies	3 copies	4 copies	6 copies	Total
Abnormal	2 (100.0%)	5 (26.3%)	3 (23.1%)	1 (100.0%)	11 (31.4%)
Normal	0 (0%)	13 (68.4%)	5 (38.5%)	0 (0%)	18 (51.4%)
NA	0 (0%)	1 (5.3%)	5 (38.5%)	0 (0%)	6 (17.1%)
Total	2 (100.0%)	19 (100.0%)	13 (100.0%)	1 (100.0%)	35 (100.0%)

NA, not available.

Our patient presents with hypotonia, global developmental delay, autism-like features, and mild facial dysmorphism, consistent with the classic clinical phenotype of 15q duplication syndrome. Trio-based whole-exome sequencing identified a *de novo* duplication involving the entire PWACR region with four copies (tetrasomy). SNP-based analysis suggested maternal origin. These findings are compatible with a possible idic(15) mechanism, although cytogenetic confirmation was not performed.

Dup15q syndrome lacks specific clinical manifestations and distinctive dysmorphic features which complicates clinical diagnosis (23). Our patient presented with hypotonia and developmental delay; however, the diagnosis of Dup15q syndrome was only confirmed by whole-exome sequencing at 1 year of age after a poor response to rehabilitation. With advancing age, continued surveillance is warranted for potential complications, including epilepsy and behavioral disorders. The characteristic malformations associated with Dup15q syndrome primarily manifest as mild nonspecific facial anomalies, such as an upturned nose, epicanthal folds, and downslanting palpebral fissures (24, 25). Notably, the affected child in our study exhibited frontal bossing and scaphocephaly, which have not been well characterized in previously reported cases.

Individuals with Dup15q syndrome show marked phenotypic variability, ranging from asymptomatic carriers to severely affected individuals. Notably, substantial heterogeneity may occur even among family members carrying the same duplication. In a three-generation pedigree, 12 individuals carried an interstitial 15q11-q13 duplication. Two first-generation females with paternally inherited duplications were phenotypically normal, whereas all 10 affected individuals in the second and third generations carried maternally inherited duplications and displayed variable phenotypes, ranging from mild developmental delay to severe manifestations, including intellectual disability, behavioral problems, and social interaction difficulties (26). This CNV may show incomplete penetrance. Isles et al. (27) estimated that maternally inherited 15q11.2-q13.1 duplications showed a penetrance of 50.5% for developmental delay/ASD/multiple congenital anomalies, approximately 2.5 times that of paternally inherited duplications (20.7%). Alternatively, the CNV may show complete penetrance with variable expressivity, such that mild or subtle clinical features may be underrecognized and misclassified as phenotypically normal (6).

The worldwide prevalence of 15q duplication syndrome is estimated to be 1 in 30,000 (2), but underdiagnosis is common due to variable expressivity, insufficient recognition, and disparities in healthcare access and resources; thus, the true prevalence is likely higher. Parijs et al. (28) identified 23 15q11-q13 duplications in 333,187 pregnant women through NIPS in 2024, corresponding to a prevalence of 1 in 14,493. Moreover, among individuals with developmental delay, intellectual disability, symptoms related to ASD or multiple congenital anomalies, the prevalence has been reported as high as 1 in 508 (29).

The duplicated interval in Dup15q syndrome encompasses several genes critical for brain development and synaptogenesis, including ubiquitin protein ligase E3A (UBE3A), small nuclear ribonucleoprotein polypeptide N (SNRPN), and three gamma-aminobutyric acid type-A (GABA_A_) receptor genes (GABRA5, GABRB3, and GABRG3) (30). Elamin et al. (31) showed that antisense oligonucleotide (ASO)-mediated reduction of UBE3A expression in Dup15q neurons prevented or ameliorated abnormal cellular phenotypes, supporting the therapeutic potential of ASO-based disease-modifying strategies. Likewise, Nakagawa et al. (32) demonstrated that a negative allosteric modulator of *α*5-containing GABA_A_ receptors improved abnormal social behaviors and cognitive inflexibility in a 15q dup mouse model, while partially normalizing aberrant electroencephalographic phenotypes. In addition, basmisanil, a GABA_A_-*α*5 negative allosteric modulator, had entered human clinical trials (NCT05307679) (32). Despite these encouraging findings, current management of Dup15q syndrome remains largely limited to symptomatic treatment and rehabilitation (33). Given the substantial impact of developmental delay and intellectual disability on quality of life, as well as the long-term burden on families and society, early identification and prenatal diagnosis hold significant clinical importance.

Previously reported prenatal cases of Dup15q syndrome generally lack specific prenatal signs and are most often identified only after invasive prenatal testing prompted by advanced maternal age or abnormal prenatal screening results (6, 12). In the present study, we collected 35 prenatal cases with Dup15q, and advanced maternal age was observed in 48.6%, consistent with previous reports linking Dup15q to higher maternal age at conception (2, 23). In addition, 22.2% of cases had a positive family history or adverse pregnancy history; given the 50% transmission risk of an interstitial duplication to offspring (28), family history and pregnancy history remain important considerations in prenatal risk assessment and genetic counseling.

In both previously reported prenatal cases and our case, FGR was consistently detected in the second trimester, and polyhydramnios in the reviewed cases was likewise identified during this period. Interestingly, both Case 13 and Case 14 showed isolated FGR, and their duplicated segments differed by only 1 base pair. Other prenatal ultrasound abnormalities included cardiac abnormalities, dysmorphic features, and abnormal soft markers. Similar findings were reported by Bisga et al. (34), who described FGR and congenital heart disease in prenatal cases. Based on the pooled analysis of prenatal cases, we provided an overview of the prenatal ultrasound spectrum of Dup15q syndrome. Although no ultrasound features specific to Dup15q syndrome were identified, nonspecific findings such as FGR and polyhydramnios may serve as clinical clues warranting further etiological evaluation. In clinical practice, cases of unexplained FGR or polyhydramnios, especially when accompanied by other abnormalities, should prompt comprehensive evaluation of underlying causes, with genetic testing considered when clinically appropriate.

Among cases with available parental origin information (*n* = 21), as shown in Table 3, the proportion of TOP was higher in maternal-origin cases (70.6%, 12/17) than in paternal-origin cases (25.0%, 1/4), suggesting that parental origin may influence pregnancy decision-making, potentially due to differences in the perceived severity of postnatal outcomes. Among live-born cases with available follow-up, the two patients with reported clinical abnormalities both carried maternal 4-copy gains of 15q11-q13 and exhibited overlapping neurodevelopmental features, including hypotonia, developmental delay, and autism-related features. In contrast, no abnormalities were reported in paternal-origin cases or those with lower duplication copy numbers (Tables 3, 4). Although limited by the small number of cases, these findings are consistent with previous evidence suggesting that parental origin and duplication copy number may contribute to phenotypic severity in Dup15q syndrome.

**Table 3:** Pregnancy and postnatal outcomes according to parental origin.

Outcomes	Parental origin			
	Maternal	Paternal	NA	Total
Pregnancy outcomes				
Live birth	5 (29.4%)	3 (75.0%)	4 (28.6%)	12 (34.3%)
TOP	12 (70.6%)	1 (25.0%)	8 (57.1%)	21 (60.0%)
NA	0 (0%)	0 (0%)	2 (14.3%)	2 (5.7%)
Postnatal clinical outcomes among live births^a^				
No reported abnormalities^b^	1 (20.0%)	3 (100%)	2 (50.0%)	6 (50.0%)
Clinical abnormalities	2 (40.0%)	0 (0%)	0 (0%)	2 (16.7%)
NA	2 (40.0%)	0 (0%)	2 (50.0%)	4 (33.3%)

NA, not available; TOP, termination of pregnancy. ^a^Percentages in the postnatal outcome section were calculated among live-born cases. ^b^No reported clinical abnormalities during the available follow-up period.

**Table 4:** Pregnancy and postnatal outcomes according to 15q11-q13 copy number.

Outcomes	15q11-q13 copy number				
	2–3 copies	3 copies	4 copies	6 copies	Total
Pregnancy outcomes					
Live birth	1 (50.0%)	8 (42.1%)	3 (23.1%)	0 (0%)	12 (34.3%)
TOP	1 (50.0%)	11 (57.9%)	8 (61.5%)	1 (100%)	21 (60.0%)
NA	0 (0%)	0 (0%)	2 (15.4%)	0 (0%)	2 (5.7%)
Postnatal clinical outcomes among live births^a^					
No reported abnormalities^b^	1 (100%)	5 (62.5%)	0 (0%)	N/A	6 (50.0%)
Clinical abnormalities	0 (0%)	0 (0%)	2 (66.7%)	N/A	2 (16.7%)
NA	0 (0%)	3 (37.5%)	1 (33.3%)	N/A	4 (33.3%)

NA, not available; TOP, termination of pregnancy. N/A, not applicable. ^a^Percentages in the postnatal outcome section were calculated among live-born cases. ^b^No reported clinical abnormalities during the available follow-up period.

In our pooled prenatal cases, the proportion of abnormal prenatal ultrasound findings was comparable between maternal- and paternal-origin cases (41.2% vs. 50.0%, Table 5). However, this observation requires further validation in larger cohorts due to the limited number of paternal-origin cases. It should be noted that a considerable proportion of families opted for TOP, which often occurred before detailed fetal structural anomaly scan or further prenatal evaluation for chromosomal copy-number abnormalities. Therefore, the detection rate of ultrasound abnormalities reported in the literature may be systematically underestimated. This methodological consideration has important implications for the design and interpretation of future studies on prenatal phenotypes. Furthermore, in maternal-origin Case 7, despite comprehensive ultrasound evaluations, facial dysmorphism was not recognized until induction at 30 + 6 weeks’ gestation, further highlighting the limited sensitivity of prenatal ultrasound for detecting subtle or nonspecific features.

**Table 5:** Prenatal ultrasound findings according to parental origin.

Prenatal ultrasound findings	Parental origin			
	Maternal	Paternal	NA	Total
Abnormal	7 (41.2%)	2 (50.0%)	2 (14.3%)	11 (31.5%)
Normal	10 (58.8%)	2 (50.0%)	6 (42.9%)	18 (51.4%)
NA	0 (0%)	0 (0%)	6 (42.9%)	6 (17.1%)
Total	17 (100.0%)	4 (100.0%)	14 (100.0%)	35 (100.0%)

NA, not available.

Prior studies have reported that NIPS has a relatively high positive predictive value for detecting such copy-number variants (6, 28). In our study, 57.1% (20/35) of cases were initially identified following a positive NIPS result. However, it is important to note that NIPS is primarily based on cell-free DNA analysis from maternal peripheral blood and is mainly designed for the detection of common aneuploidies and selected copy-number variants, rather than comprehensive genome-wide CNV analysis. Therefore, NIPS should not be considered a confirmatory diagnostic test. Although previous studies and our findings suggest that NIPS may facilitate the prenatal identification of Dup15q syndrome, a negative result cannot completely exclude this condition.

In our case, the parents’ age at conception was relatively advanced (maternal age, 34 years; paternal age, 37 years) and prenatal ultrasonography revealed abnormalities. Unfortunately, the family declined further evaluation, such as non-invasive prenatal screening and amniocentesis, highlighting the need for clearer counseling on screening options, potential benefits and risks, and informed decision-making.

A previous study reviewed cases diagnosed with 15q11-q13 duplication syndrome from 1993 to 2023, among which only nine prenatal cases were reported (34). Accordingly, the available evidence provides limited clinical guidance for prenatal diagnosis, genetic counseling, and pregnancy-related decision-making in this condition. The copy number of the duplicated segment, its parental origin, and the duplication structure can provide useful information for genetic counseling (35). However, due to the syndrome's variable expressivity and broad phenotypic spectrum, genetic counseling should emphasize individualized assessment. When selecting prenatal testing strategies and counseling families after an abnormal result, clinicians should also consider the risk of false-positive findings, parental anxiety, and the potential additional risks and burdens associated with invasive diagnostic procedures (16).

## 5 Conclusions

In conclusion, from a practical perspective, infants with early-onset hypotonia, global developmental delay, autistic-like behavioral manifestations and unsatisfactory response to standardized rehabilitation should raise suspicion for underlying genetic disorders, including Dup15q syndrome, and undergo genetic testing. Given the current lack of disease-specific treatment, prenatal screening and diagnosis are of considerable clinical importance. When isolated FGR or polyhydramnios is detected on prenatal ultrasound, routine etiological evaluation should be performed. When FGR or polyhydramnios is accompanied by additional ultrasound abnormalities (structural anomalies or sonographic soft markers), chromosomal microarray analysis may be considered. When FGR or polyhydramnios is associated with positive NIPS results or a positive family history, CNV-related disorders, including Dup15q syndrome, should be considered.

The main contributions of this study include: (1) expanding the prenatal-to-postnatal phenotypic spectrum of Dup15q syndrome by integrating the present case with previously reported prenatal cases; (2) suggesting that FGR or polyhydramnios accompanied by additional abnormalities may warrant further etiological evaluation; and (3) exploring the potential associations of parental origin and duplication copy number with pregnancy outcomes and clinical manifestations to provide insights into phenotypic variability and genetic counseling. Existing reports on 15q duplication syndrome primarily focus on postnatal cases, whereas prenatal cases remain limited in number and are often lacking in systematic and detailed prenatal clinical data. Future studies should further accumulate the reporting of prenatal cases to improve understanding of the prenatal phenotype and screening clues of this syndrome, increase prenatal detection, and provide stronger clinical support for genetic counseling.

## Data Availability

The datasets presented in this article are not readily available because of ethical and privacy restrictions. Requests to access the datasets should be directed to the corresponding authors.
