# Supplementary material for: Case Report: Prenatal and postnatal clinical manifestations of 15q duplication syndrome: a single longitudinal case combined with pooled analysis of 35 prenatal cases
Source: Front Med (Lausanne). 2026 Oct 15;13:1928018. doi: 10.3389/fmed.2026.1928018 (PMC13716719; doi:10.3389/fmed.2026.1928018)
Supplement: Supplementary file 1 [file Table1.docx]

**Table S1. Clinical information of prenatal cases with 15q duplication syndrome reported in the literature and our study.**

| **case** | **Country** | **Trimester** | **Parental origin** | **Inheritance** | **Karyotype** | **The CNVs results** | **Structural type** | **Advanced maternal age** | **Prenatal screening methods** | **Prenatal ultrasound** | **Family history** | **Pregnancy outcomes** |
| --- | --- | --- | --- | --- | --- | --- | --- | --- | --- | --- | --- | --- |
| 1 | China | T2 | Maternal | De novo | NA | seq[GRCh38] 15q11.1q13.1(19964676_28712359)x4 | NA | No | Normal prenatal serological screening | Polyhydramnios, an abdominal circumference below the 10th percentile, and mild tricuspid regurgitation | The pregnant woman and her husband were phenotypically normal. | Born, developmental delay |
| 2 | China | T1 | Maternal | De novo | 46,XX | arr[GRCh38] 15q11.2q13.1(23387530_28281759)x3* | NA | No | High risk of NIPS | A normal NT scan | The pregnant woman and her husband were phenotypically normal. | TOP at 18 weeks |
| 3 | China | T1 | Maternal | De novo | 47,XY,+mar | arr[GRCh38] 15q11.2q13.1(23123714_28399432)x4* | NA | Yes | High risk of NIPS | A normal NT scan | The pregnant woman and her husband were phenotypically normal. | TOP at 19 weeks |
| 4 | China | T1 | Maternal | De novo | 47,XX,+mar | seq[GRCh38] 15q11.2q13.2(221245102_30081994)x4* | NA | Yes | High risk of NIPS | Increased NT (3.8 mm) noted at week 12 | The pregnant woman and her husband were phenotypically normal. | TOP at 18 weeks |
| 5 | China | T2 | Maternal | NA | NA | rsa[GRCh38] 15q11q13(19794747_33307798)x2~3* | interstitial duplication | No | High risk of NIPS, abnormal prenatal serological screening results | VSD | NA | TOP |
| 6 | China | T2 | Maternal | NA | NA | arr[GRCh38] 15q11.2q13.1(23123714_28697883)x4* | NA | Yes | High risk of NIPS | A retromembrane hematoma and an umbilical cord around the neck | NA | TOP |
| 7 | China | T2 | Maternal | Maternal | 46,XX | arr[GRCh38] 15q11.2q13.1(23123714-28281759)x3* | interstitial duplication | Yes | NA | Polyhydramnios; renal pelvis separation | The pregnant woman and her son carried a 15q11-q13 duplication. She was phenotypically normal, and her son had ASD. | TOP at 30+6 weeks；facial dysmorphology noted after induction. |
| 8 | China | T2 | Maternal | Maternal | 46,XX | seq[GRCh38] 15q11.2q13.1(23374853_28194854)x3 | NA | No | High risk of NIPS | Normal | The pregnant woman and her mother carried a 15q11-q13 duplication and were phenotypically normal. | TOP |
| 9 | China | T2 | Maternal | Maternal | 46,XX | arr[GRCh38] 15q11.2q13.1(23123715_28315518)x3 | NA | No | High risk of NIPS | Normal | The pregnant woman carried a 15q11-q13 duplication and was phenotypically normal. | TOP |
| 10 | China | T2 | Maternal | Maternal | 46,XX | arr[GRCh38] 15q11.2q13.1(23387531_28302264)x3 | NA | No | High risk of NIPS | Normal | The pregnant woman and her husband were phenotypically normal. | Born, but lost to follow-up |
| 11 | China | T2 | Maternal | Maternal | 46,XX | arr[GRCh38] 15q11.2q13.1(23123715_28281759)x3 | NA | No | High risk of NIPS | Normal | The pregnant woman, her sister, and her mother carried a15q11-q13 duplication and were phenotypically normal. | Born, but lost to follow-up |
| 12 | China | T2 | Maternal | De novo | 46,XX | arr[GRCh38] 15q11.2q13.1(22582283_28769879)x3 | NA | Yes | NA | Normal | The pregnant woman and her husband were phenotypically normal. | TOP |
| 13 | China | T2 | Maternal | Maternal | 46,XX | arr[GRCh38] 15q11.2q13.1(23649402_27862008)x3* | NA | Yes | NA | FGR | The pregnant woman and her husband were phenotypically normal. | TOP at 26 weeks |
| 14 | China | T2 | Maternal | Maternal | 46,XX | arr[GRCh38] 15q11.2q13.1(23649403_27862009)x3* | NA | Yes | NA | FGR | The pregnant woman and her husband were phenotypically normal. | TOP at 26 weeks |
| 15 | China | T2 | Maternal | De novo | 46,XX,+mar | arr[GRCh38] 15q11.2q13.3(23123714_32147049)x4* | NA | No | High risk of NIPS | Normal | The pregnant woman and her husband were phenotypically normal. They had a son who died at 3 months for unknown reasons. | TOP at 24 weeks |
| 16 | China | T2 | Maternal | NA | 47,XY,+mar[13]/46,XY[9] | arr[GRCh38] 15q11.2q13.1(23123714_28446314)x3* | inv dup(15) | Yes | NA | Normal | NA | Born; followed up to 3 months of age, with normal development. |
| 17 | China | T2 | Maternal | NA | 47,XY,+inv dup(15)(pter→q13::q13→pter) | arr 15q11.1q13.2(20686219_30390043)x4, 15q13.2q13.3(30390043_32445226)x3 (genome build NA) | inv dup(15) | Yes | NA | Normal | The pregnant woman and her husband were phenotypically normal. | Born; hypotonia, ataxic gait, developmental delay, intellectual disability, epilepsy, poor speech, and autism at 21 years of age. |
| 18 | China | T2 | Paternal | NA | 46,XX | rsa[GRCh38] 15q11q13(19794747_33307798)x3* | interstitial duplication | No | High risk of NIPS, abnormal prenatal serological screening results | Bilateral choroidal cysts, absent nasal bone | NA | Born; followed up to 1 year of age, with normal development. |
| 19 | China | T2 | Paternal | Paternal | 46,XX | arr[GRCh38] 15q11.2q13.1(23123715_28281759)x3 | NA | No | NA | VSD | The pregnant woman and her husband were phenotypically normal. | Born; followed up to 6 month of age, phenotypically normal. |
| 20 | China | T2 | Paternal | De novo | 46,XX | arr[GRCh38] 15q11.2q13.1(23455960_28294829)x3 | NA | No | High risk of NIPS | Normal | The pregnant woman and her husband were phenotypically normal. | TOP |
| 21 | China | T2 | Paternal | Paternal | 46,XX | arr[GRCh38] 15q11.2q13.1(23123714_28683583)x3* | NA | No | High risk of Down syndrome screening test | Normal | The pregnant woman and her husband were phenotypically normal. | Born; followed up to 3years of age, with normal development. |
| 22 | China | T3 | NA | De novo | NA | arr[GRCh38] 15q11.2q13.3(23123714_32093613)x2~3* | interstitial duplication | No | NA | Mild fetal cerebral ventriculomegaly;  echogenic intracardiac focus;  tricuspid regurgitation | History of abortion. | Born, followed up to 3years of age, with normal development. |
| 23 | China | T2 | NA | De novo | 48,XN,+mar,+idic(15)(q13)[33]/47,XN,+idic(15)(q13)[17] | arr[GRCh38] 15q11.2q13.2(22770422_30913573)x6,15q13.2q13.3(30913574_32915089)x4 | idic(15) | No | NA | The left hand polydactyly, polyhydramnios and  FGR | The pregnant woman and her husband were phenotypically normal. | TOP at 27+5 weeks |
| 24 | China | T2 | NA | NA | 46,XX | arr[GRCh38] 15q11.2q13.1(23123715_28289312)x3 | NA | No | High risk of NIPS | Normal | The pregnant woman walked at 19 months, spoke at 2 years, and now had normal intelligence. | Born; rolled over at 6 months, sat at 9 months, crawled at 10 months, walked at 13 months, and spoke at 18 months. |
| 25 | China | T2 | NA | Paternal | 46,XX | arr[GRCh38] 15q11.2q13.3(23123715_31118640)x3 | NA | No | NA | Normal | History of adverse pregnancy outcomes; the pregnant woman and her husband were phenotypically normal. | TOP |
| 26 | China | T2 | NA | De novo | 47, XN,+psu idic(15)(q13.3) | arr[GRCh38] 15q11.2q13.3(22582283_32623522)x4 | psu idic(15) | No | High risk of NIPS | Normal | The pregnant woman and her husband were phenotypically normal. | TOP |
| 27 | Netherlands | NA | NA | NA | NA | arr[GRCh38] 15q11.2q13.1(23656946_28544359)x3 | interstitial duplication | Yes | High risk of NIPS | Normal | NA | Born |
| 28 | Netherlands | NA | NA | NA | NA | arr[GRCh38] 15q11.2(23387529_28460135)x3* | interstitial duplication | Yes | High risk of NIPS | NA | NA | TOP at 20+5 weeks |
| 29 | China | T2 | NA | NA | 47,XY, + idic(15)(q13.3) | arr[GRCh38] 15q11.2q13.3(23123714_32623522)x4* | idic(15) | Yes | NA | NA | NA | NA |
| 30 | China | T2 | NA | NA | 47,XX, + idic(15)(q13.3) | arr[GRCh38] 15q11.2q13.3(23123714_32623522)x4* | idic(15) | No | NA | NA | Adverse pregnancy history | Born |
| 31 | China | T2 | NA | NA | 47,XY, + idic(15)(q13.3) | arr[GRCh38] 15q11.2q13.3(23123714_32152060)x4* | idic(15) | Yes | High risk of NIPS | NA | NA | NA |
| 32 | China | T2 | NA | De novo | 47,XX,+mar | arr 15q11.2q13.3(18421386_29206058)x3 (genome build NA) | NA | Yes | NA | Normal | The pregnant woman and her husband were phenotypically normal. | TOP |
| 33 | China | T2 | NA | De novo | 47,XY,+psu idic(15)(q13.2) | arr[GRCh38] 15q11.2q13.2(23123714_30806488)x4 | idic(15) | Yes | High risk of NIPS | NA | The pregnant woman and her husband were phenotypically normal. | TOP |
| 34 | China | T2 | NA | NA | 47,XX,+psu idic(15)(q13.3) | arr[GRCh38] 15q11.2q13.3(23123714_32623522)x4 | idic(15) | Yes | High risk of NIPS | NA | The pregnant woman and her husband were phenotypically normal. | TOP |
| 35 | China | T2 | NA | De novo | 47,XY,+psu idic(15)(q13.3) | arr[GRCh38] 15q11.2q13.1(23123714_28315518)x4, arr[GRCh38] 15q13.1q13.3(28763894_32151842)x3 | idic(15) | Yes | High risk of NIPS | Normal | The pregnant woman and her husband were phenotypically normal. | TOP |

ASD, autism spectrum disorder; FGR, fetal growth restriction; NA, not available; NIPS, non-invasive prenatal screening; NT, nuchal translucency; T1, first trimester; T2, second trimester; T3, third trimester; TOP, termination of pregnancy; VSD, ventricular septal defect. *Coordinates were converted to GRCh38 using UCSC LiftOver (https://genome.ucsc.edu/cgi-bin/hgLiftOver).
